# Chlorido(12,17-dieth­oxy­carbonyl-11,18-dimethyl-2:3,6:7-dibutano­corrphycenato-κ^4^ *N*)iron(III)

**DOI:** 10.1107/S1600536811051130

**Published:** 2011-11-30

**Authors:** Yoshiki Ohgo, Yuki Yokoyama, Saburo Neya, Mikio Nakamura

**Affiliations:** a Department of Chemistry, Faculty of Medicine, Toho University, Ota-ku Tokyo 143-8540, Japan; b Department of Physical Chemistry, Graduate School of Pharmaceutical, Sciences, Chuoh-Inohana, Chiba, Chiba 260-8675, Japan

## Abstract

The title complex, [Fe(C_36_H_36_N_4_O_4_)Cl], shows a domed structure with a slightly distorted trapezoidpyramidal core, in which the perpendicular displacements of the Fe^III^ atom from the mean pyrrole N_4_ plane are 0.418 (3) and 0.465 (3) Å for the two crystallographically independent mol­ecules.

## Related literature

For some related metal corrphycene compounds, see: Sessler *et al.* (2000 ▶). For the structures of five-coordinated halide ligated iron(III) porphyrin, porphycene and corrphycene complexes, see: Ohgo, Neya, Funasaki *et al.* (2001 ▶); Ohgo, Neya, Ikeue *et al.* (2001 ▶); Ohgo *et al.* (2002 ▶). The surface area within the N_4_ coordinating core is significantly smaller than the corresponding area in the dianion of 2,3,7,8,12,13,17,18-octa­ethyl­porphyrin (Senge *et al.*, 1997 ▶). For the synthesis of the starting materials, see: Neya *et al.* (1998 ▶); Hombrecher & Horter (1992 ▶). Insertion of iron was carried out after Adler *et al.* (1970 ▶).
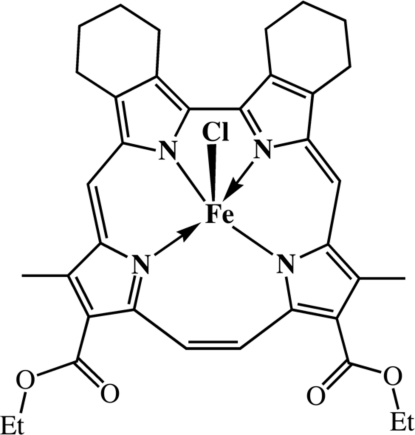


## Experimental

### 

#### Crystal data

[Fe(C_36_H_36_N_4_O_4_)Cl]
*M* *_r_* = 679.99
Triclinic, 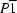

*a* = 14.455 (2) Å
*b* = 15.876 (2) Å
*c* = 16.013 (2) Å
α = 87.156 (3)°
β = 65.645 (3)°
γ = 71.291 (3)°
*V* = 3156.3 (7) Å^3^
*Z* = 4
Mo *K*α radiation
μ = 0.61 mm^−1^
*T* = 293 K
0.20 × 0.05 × 0.05 mm

#### Data collection

Rigaku R-AXIS RAPID II diffractometer
Absorption correction: multi-scan (*ABSCOR*; Higashi *et al.*, 1995 ▶) *T*_min_ = 0.888, *T*_max_ = 0.970
30087 measured reflections
14011 independent reflections
5731 reflections with *I* > 2σ(*I*)
*R*_int_ = 0.102

#### Refinement

*R*[*F* ^2^ > 2σ(*F* ^2^)] = 0.097
*wR*(*F* ^2^) = 0.305
*S* = 0.93
14011 reflections
869 parameters
H-atom parameters constrained
Δρ_max_ = 1.01 e Å^−3^
Δρ_min_ = −0.97 e Å^−3^

### 

Data collection: *CrystalClear* (Rigaku, 2002 ▶); cell refinement: *HKL-2000* (Otwinowski & Minor, 1997 ▶); data reduction: *HKL-2000*; program(s) used to solve structure: *SIR2004* (Burla *et al.*, 2005 ▶); program(s) used to refine structure: *SHELXL97* (Sheldrick, 2008 ▶); molecular graphics: *SV* (Nemoto & Ohashi, 1993 ▶) and *ORTEP* (Johnson, 1965 ▶); software used to prepare material for publication: *SHELXL97*.

## Supplementary Material

Crystal structure: contains datablock(s) I, global. DOI: 10.1107/S1600536811051130/ds2159sup1.cif

Structure factors: contains datablock(s) I. DOI: 10.1107/S1600536811051130/ds2159Isup2.hkl

Supplementary material file. DOI: 10.1107/S1600536811051130/ds2159Isup3.cdx

Additional supplementary materials:  crystallographic information; 3D view; checkCIF report

## Figures and Tables

**Table 1:** Selected geometric parameters (Å, °)

Fe1—N2	2.032 (5)
Fe1—N1	2.048 (5)
Fe1—N3	2.086 (5)
Fe1—N4	2.087 (5)
Fe1—Cl1	2.244 (2)
Fe2—N6	2.033 (6)
Fe2—N5	2.063 (5)
Fe2—N8	2.076 (5)
Fe2—N7	2.104 (5)
Fe2—Cl2	2.224 (2)

**Fig. 1.**
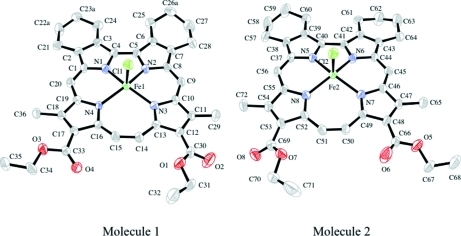
The molecular structure (ORTEP; Johnson, 1965) of the title compound with atomic numbering. Displacement ellipsoids are shown at the 30% probability level. Hydrogen atoms are omitted for clarity.

**Fig. 2.**
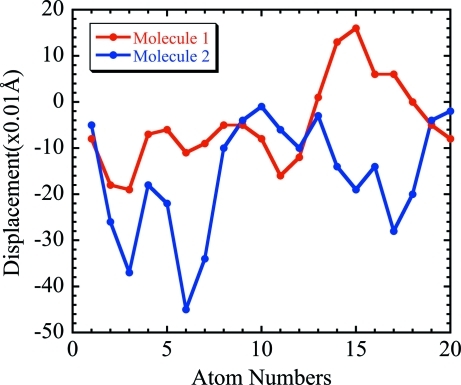
Perpendicular displacements of the peripheral 20 carbon atoms from the mean plane of 4 pyrrole nitrogen atoms.

**Fig. 3.**
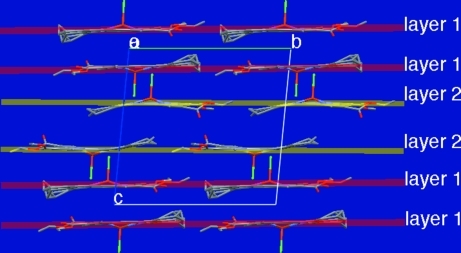
Packing diagram viewed along a axis.

## supplementary crystallographic information

### Comment

Corrphycene is one of the porphyrin isomers and it is transcribed as [18]porphyrin-(2.1.0.1). Elucidation of the structural features of iron corrphycene complexes are quite important, because they are expected to have quite unusual trapezoidal core geometry, while porphyrin complexes have square core geometry. Since the physicochemical properties of the iron(III) porphyrin complexes are affected by orbital interactions between iron and porphyrin macrocycle, the iron(III) corrphycene complexes with the distorted core should express novel physicochemcal properties. Althogh the structure of various metallocorrphycenes have been reported (Sessler *et al.*, 2000), only a few examples are reported on the structure of iron corrphycene (Ohgo *et al.*, 2002). In this paper, we report crystallographic analysis of iron(III) corrphycene complex bearing cyclohexyl rings at pyrrole β-positions.

The molecular structure of title compound with numbering is shown in Fig. 1. There are two crystallographycally independent molecules in the crystal. The corrphycene ring exhibits domed distrotion mode where the peripheral 20 carbon atoms are deviated opposit side to the iron(III) atom from the mean pyrrole N_4_ core. The central N_4_ cavity shows trapezoidal geometry with N1···N2=2.460, N2···N3=2.785, N3···N4=3.331, and N4···N1=2.799Å for molecule 1 and N5···N6=2.467, N6···N7=2.792, N7···N8=3.300, and N8···N5=2.800Å for molecule 2. Thus, the surface area within the N_4_ coordinating core are 7.986 Å^2^ and 7.973 Å^2^, which are significantly smaller than the corresponding area of iron porphyrins; 8.123 Å^2^ in [Fe(OEP)Cl](OEP: dianion of 2,3,7,8,12,13,17,18- octaethylporphyrin, Senge *et al.*, 1997). The axial Fe—Cl bond distances are 2.244 (2)Å and 2.225 (2) Å, which are comparable to that reported for [Fe(OEP)Cl], 2.231 Å. The Fe—N_p_ bond distances for direct-linked pyrroles, 2.049 (5) and 2.031 (5)Å for molecule 1 and 2.065 (5) and 2.035 (5)Å for molecule 2, are slightly shorter than those for etheno-bridged pyrroles, 2.086 (5) and 2.086 (5)Å for molecule 1 and 2.103 (5) and 2.076 (5)Å for molecule 2; the average Fe—N_p_ bond distance is 2.07Å in [Fe(OEP)Cl]. The deviation of the central iron(III) atom from the least-squares plane of the 4 pyrrole N atoms, 0.418 (3)Å for molecule 1 and 0.465 (3)Å for molecule 2, are significantly smaller than that in [Fe(OEP)Cl], 0.495 Å. Interestingly, the distortion degree of the macrocycles and perpendicular displacement of the iron(III) atoms are different between molecule 1 and 2, suggesting that the the contribution of the S=3/2 spin state in molecular 1 could be larger than that in the molecule 2(Fig.2). These differences in the cavity geometries between iron(III) corrphycenes and iron(III) porphyrins should be reflected in the physicochemical properties. Fig. 3 shows a packing diagram viewed along *a* axis. The molecule 1 and molecule 2 form a counterpart making layers in the *ab* plane. The two layers consist of the molecule 1(layer 1) and the two layers consist of molecule 2(layer 2) stack alternately. The distance between the closest homo-layers for layer 1 and layer 2 are detemined to be 3.693Å and 4.400 Å, respectively.

### Experimental

Ethyl 4,5,6,7-tetrahydro-2*H*-isoindole-1-carboxylate was prepared from 2-formyl cyclohexanone and ethyl glycine hydrochloride according to the reported method (Hombrecher *et al.*, 1992). The compound was derived into 2:3,6:7-dibutano-12,17-diethoxycarbonyl-11,18-dimethyl-corrphycene according to the reported method (Neya *et al.*, 1998). Insertion of iron was carried out after Adler (Adler *et al.*, 1970). The solid was recrystallized from chloroform solution.

### Refinement

There are disorders in the two cyclohexyl groups at pyrrole β-position. The site occupation factor for main groups of disordered groups are 0.63 and 0.52, respectively. H atoms were refined using a riding model. H atoms were refined using a riding model. The positional parameters of H atoms were constrained to have the C—H distances of 0.96Å for primary, 0.97Å for secondary, and 0.93Å for aromatic. Hydrogen U values constrained to 1.2 times the equivalent isotropic U of the atoms to which they are attached (1.5 for methyl groups).

### Crystal data

**Table:**

[Fe(C_36_H_36_N_4_O_4_)Cl]	*Z* = 4
*M**_r_* = 679.99	*F*(000) = 1420
Triclinic, *P*1	*D*_x_ = 1.431 Mg m^−^^3^
Hall symbol: -P 1	Mo *K*α radiation, λ = 0.71073 Å
*a* = 14.455 (2) Å	Cell parameters from 30087 reflections
*b* = 15.876 (2) Å	θ = 3.0–27.5°
*c* = 16.013 (2) Å	µ = 0.61 mm^−^^1^
α = 87.156 (3)°	*T* = 293 K
β = 65.645 (3)°	Needle, purple
γ = 71.291 (3)°	0.20 × 0.05 × 0.05 mm
*V* = 3156.3 (7) Å^3^	

### Data collection

**Table:**

Rigaku R-AXIS RAPID II diffractometer	14011 independent reflections
Radiation source: fine-focus sealed tube	5731 reflections with *I* > 2σ(*I*)
graphite	*R*_int_ = 0.102
Detector resolution: 10 pixels mm^-1^	θ_max_ = 27.5°, θ_min_ = 3.0°
ω scans	*h* = −16→18
Absorption correction: multi-scan (*ABSCOR*; Higashi *et al.*, 1995)	*k* = −20→20
*T*_min_ = 0.888, *T*_max_ = 0.970	*l* = −20→20
30087 measured reflections	

### Refinement

**Table:**

Refinement on *F*^2^	Primary atom site location: structure-invariant direct methods
Least-squares matrix: full	Secondary atom site location: difference Fourier map
*R*[*F*^2^ > 2σ(*F*^2^)] = 0.097	Hydrogen site location: inferred from neighbouring sites
*wR*(*F*^2^) = 0.305	H-atom parameters constrained
*S* = 0.93	*w* = 1/[σ^2^(*F*_o_^2^) + (0.1573*P*)^2^] where *P* = (*F*_o_^2^ + 2*F*_c_^2^)/3
14011 reflections	(Δ/σ)_max_ < 0.001
869 parameters	Δρ_max_ = 1.01 e Å^−^^3^
0 restraints	Δρ_min_ = −0.97 e Å^−^^3^

### Special details

**Table:**

Geometry. All e.s.d.'s (except the e.s.d. in the dihedral angle between two l.s. planes) are estimated using the full covariance matrix. The cell e.s.d.'s are taken into account individually in the estimation of e.s.d.'s in distances, angles and torsion angles; correlations between e.s.d.'s in cell parameters are only used when they are defined by crystal symmetry. An approximate (isotropic) treatment of cell e.s.d.'s is used for estimating e.s.d.'s involving l.s. planes.
Refinement. Refinement of *F*^2^ against ALL reflections. The weighted *R*-factor *wR* and goodness of fit *S* are based on *F*^2^, conventional *R*-factors *R* are based on *F*, with *F* set to zero for negative *F*^2^. The threshold expression of *F*^2^ > σ(*F*^2^) is used only for calculating *R*-factors(gt) *etc*. and is not relevant to the choice of reflections for refinement. *R*-factors based on *F*^2^ are statistically about twice as large as those based on *F*, and *R*- factors based on ALL data will be even larger.

### Fractional atomic coordinates and isotropic or equivalent isotropic displacement parameters (Å^2^)

**Table:**

	*x*	*y*	*z*	*U*_iso_*/*U*_eq_	Occ. (<1)
Fe1	0.03160 (6)	0.05195 (6)	0.15031 (5)	0.0515 (3)	
Cl1	−0.04106 (12)	0.05985 (12)	0.30472 (10)	0.0638 (4)	
N1	−0.0763 (4)	0.1525 (4)	0.1192 (3)	0.0578 (12)	
N2	0.0917 (4)	0.1546 (4)	0.1223 (3)	0.0544 (12)	
N3	0.1886 (4)	−0.0278 (3)	0.1240 (3)	0.0527 (11)	
N4	−0.0389 (3)	−0.0316 (3)	0.1205 (3)	0.0486 (11)	
O1	0.3671 (4)	−0.2988 (4)	0.1438 (4)	0.0944 (17)	
O2	0.5050 (5)	−0.2550 (5)	0.0832 (6)	0.137 (3)	
O3	−0.1935 (4)	−0.2483 (3)	0.1476 (3)	0.0738 (13)	
O4	−0.0219 (4)	−0.3116 (4)	0.1174 (5)	0.0953 (18)	
C1	−0.1694 (4)	0.1578 (4)	0.1124 (4)	0.0529 (14)	
C2	−0.2235 (5)	0.2490 (4)	0.1045 (4)	0.0586 (15)	
C3	−0.1591 (5)	0.2980 (4)	0.1046 (4)	0.0569 (14)	
C4	−0.0691 (5)	0.2365 (4)	0.1149 (4)	0.0553 (14)	
C5	0.0280 (5)	0.2383 (4)	0.1169 (4)	0.0552 (14)	
C6	0.0775 (5)	0.3041 (4)	0.1140 (4)	0.0578 (15)	
C7	0.1733 (5)	0.2574 (5)	0.1168 (4)	0.0583 (15)	
C8	0.1833 (5)	0.1657 (4)	0.1204 (4)	0.0541 (14)	
C9	0.2651 (5)	0.0922 (5)	0.1213 (4)	0.0555 (14)	
H9	0.3239	0.1031	0.1229	0.067*	
C10	0.2701 (4)	0.0034 (4)	0.1200 (4)	0.0538 (14)	
C11	0.3607 (5)	−0.0692 (4)	0.1158 (4)	0.0547 (14)	
C12	0.3346 (5)	−0.1468 (4)	0.1186 (4)	0.0538 (14)	
C13	0.2254 (5)	−0.1188 (4)	0.1258 (4)	0.0565 (15)	
C14	0.1723 (5)	−0.1811 (5)	0.1333 (5)	0.0647 (17)	
H14	0.2128	−0.2379	0.1392	0.078*	
C15	0.0765 (5)	−0.1803 (5)	0.1342 (5)	0.0634 (16)	
H15	0.0690	−0.2365	0.1441	0.076*	
C16	−0.0105 (4)	−0.1227 (4)	0.1250 (4)	0.0489 (13)	
C17	−0.0960 (5)	−0.1521 (4)	0.1238 (4)	0.0522 (13)	
C18	−0.1742 (4)	−0.0782 (4)	0.1180 (3)	0.0494 (13)	
C19	−0.1376 (5)	−0.0029 (4)	0.1154 (4)	0.0513 (13)	
C20	−0.1969 (5)	0.0830 (4)	0.1121 (4)	0.0551 (14)	
H20	−0.2622	0.0912	0.1093	0.066*	
C21	−0.3241 (5)	0.2869 (5)	0.0920 (4)	0.0652 (17)	
H21A	−0.3834	0.2802	0.1465	0.078*	
H21B	−0.3187	0.2532	0.0403	0.078*	
C22A	−0.349 (2)	0.3842 (12)	0.075 (2)	0.106 (8)	0.626 (15)
H22A	−0.3284	0.3872	0.0095	0.127*	0.626 (15)
H22B	−0.4260	0.4123	0.1056	0.127*	0.626 (15)
C23A	−0.3019 (11)	0.4372 (8)	0.1016 (12)	0.080 (4)	0.626 (15)
H23A	−0.3483	0.4607	0.1653	0.095*	0.626 (15)
H23B	−0.3033	0.4879	0.0652	0.095*	0.626 (15)
C22B	−0.320 (4)	0.367 (3)	0.049 (5)	0.19 (3)	0.374 (15)
H22C	−0.3048	0.3505	−0.0143	0.233*	0.374 (15)
H22D	−0.3945	0.4059	0.0757	0.233*	0.374 (15)
C23B	−0.268 (2)	0.4165 (15)	0.042 (2)	0.080 (4)	0.374 (15)
H23C	−0.3182	0.4766	0.0641	0.095*	0.374 (15)
H23D	−0.2241	0.4167	−0.0230	0.095*	0.374 (15)
C24	−0.1886 (6)	0.3963 (5)	0.0943 (5)	0.078 (2)	
H24A	−0.1386	0.4061	0.0349	0.093*	
H24B	−0.1814	0.4263	0.1415	0.093*	
C25	0.0372 (7)	0.4032 (5)	0.1142 (5)	0.0736 (19)	
H25A	0.0475	0.4183	0.0522	0.088*	
H25B	−0.0393	0.4256	0.1534	0.088*	
C26A	0.094 (2)	0.4472 (12)	0.148 (3)	0.079 (6)	0.52 (5)
H26A	0.0646	0.4496	0.2143	0.095*	0.52 (5)
H26B	0.0809	0.5080	0.1308	0.095*	0.52 (5)
C26B	0.129 (2)	0.4422 (14)	0.091 (3)	0.082 (9)	0.48 (5)
H26C	0.1544	0.4510	0.0259	0.099*	0.48 (5)
H26D	0.0970	0.5010	0.1243	0.099*	0.48 (5)
C27	0.2162 (9)	0.3976 (7)	0.1073 (9)	0.119 (3)	
H27A	0.2462	0.4064	0.0424	0.143*	
H27B	0.2468	0.4259	0.1368	0.143*	
C28	0.2527 (6)	0.3013 (6)	0.1158 (5)	0.0729 (19)	
H28A	0.2627	0.2918	0.1722	0.087*	
H28B	0.3216	0.2732	0.0646	0.087*	
C29	0.4628 (5)	−0.0631 (5)	0.1117 (5)	0.0706 (18)	
H29A	0.5018	−0.1182	0.1272	0.106*	
H29B	0.4472	−0.0150	0.1547	0.106*	
H29C	0.5055	−0.0523	0.0506	0.106*	
C30	0.4078 (5)	−0.2352 (5)	0.1135 (5)	0.0657 (17)	
C31	0.4369 (7)	−0.3887 (6)	0.1409 (7)	0.101 (3)	
H31A	0.4721	−0.3897	0.1812	0.121*	
H31B	0.4919	−0.4089	0.0787	0.121*	
C32	0.3722 (8)	−0.4471 (8)	0.1706 (9)	0.129 (4)	
H32A	0.3340	−0.4391	0.2365	0.194*	
H32B	0.4182	−0.5081	0.1516	0.194*	
H32C	0.3217	−0.4328	0.1434	0.194*	
C33	−0.0952 (5)	−0.2444 (5)	0.1274 (4)	0.0597 (15)	
C34	−0.2055 (7)	−0.3358 (6)	0.1559 (6)	0.084 (2)	
H34A	−0.1961	−0.3610	0.2094	0.101*	
H34B	−0.1528	−0.3759	0.1016	0.101*	
C35	−0.3158 (7)	−0.3221 (7)	0.1657 (6)	0.096 (3)	
H35A	−0.3670	−0.2883	0.2235	0.144*	
H35B	−0.3253	−0.3790	0.1638	0.144*	
H35C	−0.3268	−0.2902	0.1163	0.144*	
C36	−0.2784 (5)	−0.0695 (5)	0.1141 (5)	0.0629 (16)	
H36A	−0.2884	−0.0294	0.0693	0.094*	
H36B	−0.3365	−0.0465	0.1734	0.094*	
H36C	−0.2773	−0.1271	0.0970	0.094*	
Fe2	0.41614 (6)	0.16429 (6)	0.31906 (5)	0.0525 (3)	
Cl2	0.48664 (13)	0.15809 (13)	0.16615 (10)	0.0664 (4)	
N5	0.3520 (4)	0.0620 (3)	0.3505 (3)	0.0549 (12)	
N6	0.5216 (4)	0.0638 (4)	0.3510 (3)	0.0561 (12)	
N7	0.4892 (4)	0.2469 (3)	0.3488 (3)	0.0536 (12)	
N8	0.2588 (4)	0.2465 (3)	0.3540 (3)	0.0548 (12)	
O5	0.6166 (5)	0.4666 (4)	0.3799 (5)	0.103 (2)	
O6	0.4819 (8)	0.5228 (5)	0.3462 (8)	0.178 (4)	
O7	0.0724 (4)	0.5177 (4)	0.3472 (3)	0.0773 (14)	
O8	−0.0666 (4)	0.4765 (4)	0.4400 (4)	0.0985 (18)	
C37	0.2580 (5)	0.0560 (4)	0.3567 (4)	0.0541 (14)	
C38	0.2597 (5)	−0.0346 (5)	0.3714 (4)	0.0588 (15)	
C39	0.3545 (5)	−0.0822 (4)	0.3771 (4)	0.0584 (15)	
C40	0.4117 (5)	−0.0206 (4)	0.3636 (4)	0.0517 (13)	
C41	0.5096 (4)	−0.0183 (4)	0.3652 (4)	0.0484 (12)	
C42	0.5976 (5)	−0.0792 (4)	0.3795 (4)	0.0545 (14)	
C43	0.6661 (4)	−0.0337 (4)	0.3708 (4)	0.0530 (14)	
C44	0.6183 (4)	0.0549 (4)	0.3549 (4)	0.0519 (13)	
C45	0.6501 (5)	0.1298 (4)	0.3502 (4)	0.0545 (14)	
H45	0.7183	0.1198	0.3475	0.065*	
C46	0.5923 (4)	0.2154 (4)	0.3491 (4)	0.0548 (15)	
C47	0.6261 (5)	0.2906 (4)	0.3520 (4)	0.0533 (14)	
C48	0.5457 (5)	0.3653 (4)	0.3554 (4)	0.0587 (15)	
C49	0.4592 (5)	0.3378 (4)	0.3523 (4)	0.0558 (14)	
C50	0.3621 (5)	0.4009 (5)	0.3613 (5)	0.0666 (17)	
H50	0.3633	0.4586	0.3656	0.080*	
C51	0.2654 (5)	0.4014 (5)	0.3655 (5)	0.0707 (18)	
H51	0.2192	0.4595	0.3718	0.085*	
C52	0.2165 (5)	0.3386 (5)	0.3629 (4)	0.0576 (15)	
C53	0.1060 (5)	0.3668 (5)	0.3745 (4)	0.0602 (16)	
C54	0.0815 (5)	0.2916 (5)	0.3689 (4)	0.0600 (16)	
C55	0.1756 (4)	0.2165 (4)	0.3573 (4)	0.0532 (14)	
C56	0.1773 (5)	0.1295 (4)	0.3560 (4)	0.0549 (14)	
H56	0.1179	0.1199	0.3545	0.066*	
C57	0.1755 (6)	−0.0738 (6)	0.3841 (5)	0.076 (2)	
H57A	0.1717	−0.0791	0.3256	0.092*	
H57B	0.1065	−0.0338	0.4269	0.092*	
C58	0.1956 (8)	−0.1643 (7)	0.4200 (9)	0.125 (4)	
H58A	0.1638	−0.1558	0.4867	0.150*	
H58B	0.1583	−0.1963	0.4026	0.150*	
C59	0.3077 (9)	−0.2204 (7)	0.3886 (9)	0.118 (3)	
H59A	0.3334	−0.2429	0.3250	0.142*	
H59B	0.3106	−0.2715	0.4245	0.142*	
C60	0.3845 (6)	−0.1781 (5)	0.3939 (5)	0.0704 (18)	
H60A	0.3851	−0.1834	0.4543	0.085*	
H60B	0.4566	−0.2097	0.3485	0.085*	
C61	0.6186 (5)	−0.1758 (4)	0.4014 (4)	0.0613 (16)	
H61A	0.5545	−0.1819	0.4509	0.074*	
H61B	0.6361	−0.2136	0.3477	0.074*	
C62	0.7109 (9)	−0.2044 (7)	0.4300 (8)	0.113 (3)	
H62A	0.6797	−0.1934	0.4965	0.135*	
H62B	0.7436	−0.2687	0.4155	0.135*	
C63	0.7932 (8)	−0.1689 (7)	0.3956 (8)	0.110 (3)	
H63A	0.8444	−0.2017	0.3364	0.132*	
H63B	0.8295	−0.1813	0.4363	0.132*	
C64	0.7689 (5)	−0.0725 (5)	0.3826 (4)	0.0651 (17)	
H64A	0.8277	−0.0641	0.3287	0.078*	
H64B	0.7633	−0.0406	0.4355	0.078*	
C65	0.7305 (5)	0.2835 (5)	0.3518 (5)	0.0714 (18)	
H65A	0.7644	0.3182	0.3058	0.107*	
H65B	0.7759	0.2220	0.3383	0.107*	
H65C	0.7191	0.3055	0.4112	0.107*	
C66	0.5417 (6)	0.4584 (5)	0.3606 (5)	0.0710 (18)	
C67	0.6256 (7)	0.5543 (6)	0.3884 (7)	0.095 (3)	
H67A	0.5701	0.5877	0.4460	0.114*	
H67B	0.6183	0.5880	0.3379	0.114*	
C68	0.7342 (9)	0.5374 (8)	0.3856 (8)	0.126 (4)	
H68A	0.7493	0.4886	0.4212	0.189*	
H68B	0.7362	0.5900	0.4109	0.189*	
H68C	0.7872	0.5226	0.3230	0.189*	
C69	0.0288 (6)	0.4579 (5)	0.3905 (5)	0.0718 (19)	
C70	0.0044 (7)	0.6107 (5)	0.3625 (6)	0.086 (2)	
H70A	−0.0366	0.6203	0.3258	0.103*	
H70B	−0.0459	0.6250	0.4269	0.103*	
C71	0.0695 (10)	0.6682 (8)	0.3376 (12)	0.181 (7)	
H71A	0.1209	0.6499	0.3640	0.271*	
H71B	0.0242	0.7288	0.3605	0.271*	
H71C	0.1069	0.6640	0.2718	0.271*	
C72	−0.0205 (5)	0.2847 (6)	0.3722 (5)	0.078 (2)	
H72A	−0.0637	0.3417	0.3638	0.117*	
H72B	−0.0595	0.2666	0.4308	0.117*	
H72C	−0.0041	0.2413	0.3241	0.117*	

### Atomic displacement parameters (Å^2^)

**Table:**

	*U*^11^	*U*^22^	*U*^33^	*U*^12^	*U*^13^	*U*^23^
Fe1	0.0461 (4)	0.0481 (5)	0.0627 (5)	−0.0178 (4)	−0.0233 (3)	0.0035 (4)
Cl1	0.0575 (8)	0.0751 (11)	0.0603 (8)	−0.0266 (8)	−0.0220 (6)	0.0025 (7)
N1	0.057 (3)	0.054 (3)	0.067 (3)	−0.024 (2)	−0.026 (2)	0.008 (2)
N2	0.045 (2)	0.056 (3)	0.067 (3)	−0.023 (2)	−0.024 (2)	0.002 (2)
N3	0.050 (3)	0.050 (3)	0.061 (3)	−0.019 (2)	−0.023 (2)	0.003 (2)
N4	0.038 (2)	0.047 (3)	0.067 (3)	−0.014 (2)	−0.0283 (19)	0.004 (2)
O1	0.066 (3)	0.061 (4)	0.153 (5)	−0.009 (3)	−0.052 (3)	0.010 (3)
O2	0.065 (4)	0.087 (5)	0.243 (8)	−0.022 (3)	−0.053 (4)	0.036 (5)
O3	0.063 (3)	0.060 (3)	0.111 (3)	−0.030 (2)	−0.041 (2)	0.012 (2)
O4	0.066 (3)	0.048 (3)	0.173 (5)	−0.019 (3)	−0.050 (3)	−0.001 (3)
C1	0.041 (3)	0.055 (4)	0.067 (3)	−0.018 (3)	−0.026 (2)	0.011 (3)
C2	0.055 (3)	0.050 (4)	0.062 (3)	−0.008 (3)	−0.023 (3)	0.000 (3)
C3	0.055 (3)	0.046 (4)	0.066 (3)	−0.017 (3)	−0.022 (3)	0.009 (3)
C4	0.050 (3)	0.052 (4)	0.063 (3)	−0.016 (3)	−0.023 (2)	0.008 (3)
C5	0.047 (3)	0.052 (4)	0.064 (3)	−0.017 (3)	−0.021 (2)	0.006 (3)
C6	0.064 (4)	0.052 (4)	0.058 (3)	−0.029 (3)	−0.019 (3)	0.010 (3)
C7	0.063 (4)	0.064 (4)	0.061 (3)	−0.033 (3)	−0.030 (3)	0.007 (3)
C8	0.054 (3)	0.054 (4)	0.063 (3)	−0.027 (3)	−0.027 (2)	0.007 (3)
C9	0.047 (3)	0.065 (4)	0.063 (3)	−0.027 (3)	−0.024 (2)	0.006 (3)
C10	0.046 (3)	0.061 (4)	0.062 (3)	−0.022 (3)	−0.027 (2)	0.004 (3)
C11	0.044 (3)	0.058 (4)	0.067 (3)	−0.017 (3)	−0.028 (2)	0.007 (3)
C12	0.045 (3)	0.055 (4)	0.061 (3)	−0.012 (3)	−0.026 (2)	0.007 (3)
C13	0.047 (3)	0.056 (4)	0.062 (3)	−0.016 (3)	−0.019 (2)	−0.001 (3)
C14	0.054 (3)	0.057 (4)	0.091 (4)	−0.022 (3)	−0.035 (3)	0.003 (3)
C15	0.065 (4)	0.050 (4)	0.093 (4)	−0.029 (3)	−0.043 (3)	0.013 (3)
C16	0.044 (3)	0.040 (3)	0.062 (3)	−0.016 (2)	−0.020 (2)	0.007 (2)
C17	0.052 (3)	0.045 (3)	0.063 (3)	−0.022 (3)	−0.022 (2)	0.004 (2)
C18	0.048 (3)	0.050 (3)	0.052 (3)	−0.020 (3)	−0.019 (2)	0.004 (2)
C19	0.050 (3)	0.052 (4)	0.054 (3)	−0.018 (3)	−0.022 (2)	0.002 (2)
C20	0.052 (3)	0.058 (4)	0.064 (3)	−0.025 (3)	−0.028 (2)	0.015 (3)
C21	0.062 (4)	0.058 (5)	0.072 (4)	−0.014 (3)	−0.028 (3)	0.007 (3)
C22A	0.095 (13)	0.033 (9)	0.23 (2)	−0.029 (10)	−0.106 (14)	0.042 (13)
C23A	0.084 (8)	0.037 (6)	0.118 (11)	−0.004 (5)	−0.054 (8)	0.006 (6)
C22B	0.16 (4)	0.10 (3)	0.35 (6)	0.08 (3)	−0.21 (4)	−0.12 (4)
C23B	0.084 (8)	0.037 (6)	0.118 (11)	−0.004 (5)	−0.054 (8)	0.006 (6)
C24	0.079 (5)	0.062 (5)	0.093 (5)	−0.021 (4)	−0.038 (4)	0.015 (4)
C25	0.086 (5)	0.063 (5)	0.080 (4)	−0.034 (4)	−0.037 (4)	0.021 (3)
C26A	0.103 (14)	0.056 (9)	0.079 (14)	−0.029 (9)	−0.037 (12)	0.003 (9)
C26B	0.090 (14)	0.060 (11)	0.11 (2)	−0.046 (10)	−0.039 (14)	0.015 (12)
C27	0.117 (8)	0.090 (8)	0.179 (10)	−0.055 (7)	−0.075 (7)	0.011 (7)
C28	0.082 (5)	0.084 (6)	0.073 (4)	−0.045 (4)	−0.039 (3)	0.014 (3)
C29	0.058 (4)	0.076 (5)	0.087 (4)	−0.023 (4)	−0.039 (3)	0.016 (4)
C30	0.048 (3)	0.075 (5)	0.083 (4)	−0.025 (3)	−0.032 (3)	0.006 (3)
C31	0.066 (5)	0.061 (5)	0.179 (9)	0.003 (4)	−0.073 (5)	0.013 (5)
C32	0.082 (6)	0.092 (8)	0.210 (12)	−0.028 (6)	−0.062 (7)	0.042 (8)
C33	0.054 (3)	0.054 (4)	0.078 (4)	−0.023 (3)	−0.030 (3)	0.003 (3)
C34	0.095 (5)	0.069 (5)	0.112 (6)	−0.050 (4)	−0.050 (4)	0.011 (4)
C35	0.081 (5)	0.109 (7)	0.116 (6)	−0.053 (5)	−0.041 (4)	−0.008 (5)
C36	0.053 (3)	0.056 (4)	0.090 (4)	−0.021 (3)	−0.038 (3)	0.009 (3)
Fe2	0.0489 (5)	0.0531 (6)	0.0605 (5)	−0.0216 (4)	−0.0239 (3)	0.0051 (4)
Cl2	0.0639 (9)	0.0815 (12)	0.0609 (8)	−0.0358 (9)	−0.0242 (6)	0.0076 (7)
N5	0.050 (3)	0.053 (3)	0.066 (3)	−0.019 (2)	−0.027 (2)	0.006 (2)
N6	0.058 (3)	0.057 (3)	0.061 (3)	−0.034 (3)	−0.021 (2)	0.008 (2)
N7	0.056 (3)	0.051 (3)	0.061 (3)	−0.020 (2)	−0.029 (2)	0.008 (2)
N8	0.049 (3)	0.050 (3)	0.065 (3)	−0.016 (2)	−0.024 (2)	0.006 (2)
O5	0.116 (5)	0.059 (4)	0.180 (6)	−0.036 (3)	−0.101 (4)	0.011 (3)
O6	0.168 (8)	0.060 (5)	0.397 (15)	−0.049 (5)	−0.198 (9)	0.044 (6)
O7	0.065 (3)	0.060 (3)	0.100 (3)	−0.010 (3)	−0.036 (2)	0.011 (3)
O8	0.052 (3)	0.086 (4)	0.138 (5)	−0.015 (3)	−0.026 (3)	−0.002 (3)
C37	0.058 (3)	0.061 (4)	0.059 (3)	−0.035 (3)	−0.028 (2)	0.012 (3)
C38	0.059 (3)	0.068 (4)	0.063 (3)	−0.041 (3)	−0.024 (3)	0.009 (3)
C39	0.063 (4)	0.055 (4)	0.060 (3)	−0.027 (3)	−0.022 (3)	0.007 (3)
C40	0.049 (3)	0.048 (3)	0.061 (3)	−0.019 (3)	−0.022 (2)	0.003 (2)
C41	0.051 (3)	0.041 (3)	0.058 (3)	−0.017 (3)	−0.026 (2)	0.006 (2)
C42	0.051 (3)	0.047 (3)	0.065 (3)	−0.014 (3)	−0.025 (2)	0.007 (3)
C43	0.044 (3)	0.052 (4)	0.062 (3)	−0.008 (3)	−0.028 (2)	0.006 (2)
C44	0.044 (3)	0.055 (4)	0.065 (3)	−0.020 (3)	−0.029 (2)	0.008 (3)
C45	0.049 (3)	0.046 (4)	0.069 (3)	−0.014 (3)	−0.026 (2)	0.007 (3)
C46	0.047 (3)	0.068 (4)	0.052 (3)	−0.021 (3)	−0.022 (2)	0.002 (3)
C47	0.046 (3)	0.060 (4)	0.063 (3)	−0.025 (3)	−0.026 (2)	0.009 (3)
C48	0.060 (3)	0.056 (4)	0.077 (4)	−0.030 (3)	−0.036 (3)	0.010 (3)
C49	0.054 (3)	0.044 (3)	0.077 (4)	−0.023 (3)	−0.030 (3)	0.011 (3)
C50	0.060 (4)	0.051 (4)	0.107 (5)	−0.022 (3)	−0.050 (3)	0.016 (3)
C51	0.048 (3)	0.058 (4)	0.103 (5)	−0.012 (3)	−0.032 (3)	0.003 (4)
C52	0.043 (3)	0.063 (4)	0.068 (3)	−0.012 (3)	−0.029 (2)	0.007 (3)
C53	0.043 (3)	0.065 (4)	0.073 (4)	−0.019 (3)	−0.025 (3)	0.009 (3)
C54	0.050 (3)	0.070 (4)	0.065 (3)	−0.024 (3)	−0.026 (3)	0.007 (3)
C55	0.048 (3)	0.065 (4)	0.054 (3)	−0.030 (3)	−0.019 (2)	0.006 (3)
C56	0.047 (3)	0.055 (4)	0.074 (3)	−0.021 (3)	−0.034 (3)	0.017 (3)
C57	0.078 (5)	0.079 (5)	0.098 (5)	−0.043 (4)	−0.048 (4)	0.012 (4)
C58	0.093 (7)	0.095 (8)	0.227 (12)	−0.073 (6)	−0.080 (7)	0.059 (7)
C59	0.123 (8)	0.093 (8)	0.187 (10)	−0.067 (7)	−0.089 (8)	0.031 (7)
C60	0.074 (4)	0.064 (5)	0.081 (4)	−0.024 (4)	−0.039 (3)	0.011 (3)
C61	0.066 (4)	0.045 (4)	0.069 (4)	−0.015 (3)	−0.027 (3)	0.007 (3)
C62	0.119 (8)	0.076 (7)	0.185 (10)	−0.038 (6)	−0.103 (7)	0.043 (6)
C63	0.092 (6)	0.070 (6)	0.199 (10)	−0.031 (5)	−0.089 (7)	0.042 (6)
C64	0.059 (4)	0.070 (5)	0.067 (4)	−0.022 (3)	−0.027 (3)	0.006 (3)
C65	0.061 (4)	0.070 (5)	0.097 (5)	−0.030 (4)	−0.041 (3)	0.010 (4)
C66	0.057 (4)	0.051 (4)	0.117 (5)	−0.019 (3)	−0.045 (4)	0.006 (4)
C67	0.097 (6)	0.071 (6)	0.144 (7)	−0.051 (5)	−0.058 (5)	0.005 (5)
C68	0.128 (8)	0.101 (8)	0.198 (11)	−0.066 (7)	−0.093 (8)	0.005 (7)
C69	0.062 (4)	0.066 (5)	0.091 (5)	−0.018 (4)	−0.039 (3)	0.002 (4)
C70	0.074 (5)	0.063 (5)	0.114 (6)	−0.007 (4)	−0.045 (4)	0.003 (4)
C71	0.088 (8)	0.082 (8)	0.298 (18)	−0.009 (7)	−0.028 (9)	0.030 (10)
C72	0.051 (4)	0.085 (6)	0.105 (5)	−0.026 (4)	−0.037 (3)	0.009 (4)

### Geometric parameters (Å, °)

**Table:**

Fe1—N2	2.032 (5)	C35—H35B	0.9600
Fe1—N1	2.048 (5)	C35—H35C	0.9600
Fe1—N3	2.086 (5)	C36—H36A	0.9600
Fe1—N4	2.087 (5)	C36—H36B	0.9600
Fe1—Cl1	2.244 (2)	C36—H36C	0.9600
N1—C4	1.367 (8)	Fe2—N6	2.033 (6)
N1—C1	1.371 (7)	Fe2—N5	2.063 (5)
N2—C5	1.375 (8)	Fe2—N8	2.076 (5)
N2—C8	1.379 (7)	Fe2—N7	2.104 (5)
N3—C13	1.374 (8)	Fe2—Cl2	2.224 (2)
N3—C10	1.395 (7)	N5—C37	1.354 (7)
N4—C16	1.378 (7)	N5—C40	1.380 (7)
N4—C19	1.387 (7)	N6—C41	1.367 (8)
O1—C30	1.302 (9)	N6—C44	1.386 (7)
O1—C31	1.449 (9)	N7—C49	1.364 (8)
O2—C30	1.216 (8)	N7—C46	1.414 (7)
O3—C33	1.342 (8)	N8—C52	1.381 (8)
O3—C34	1.447 (9)	N8—C55	1.411 (7)
O4—C33	1.199 (8)	O5—C66	1.288 (8)
C1—C20	1.369 (9)	O5—C67	1.457 (10)
C1—C2	1.436 (9)	O6—C66	1.192 (10)
C2—C3	1.392 (9)	O7—C69	1.318 (9)
C2—C21	1.477 (9)	O7—C70	1.453 (9)
C3—C4	1.420 (9)	O8—C69	1.216 (9)
C3—C24	1.500 (10)	C37—C56	1.364 (8)
C4—C5	1.427 (8)	C37—C38	1.440 (9)
C5—C6	1.434 (9)	C38—C39	1.374 (9)
C6—C7	1.362 (9)	C38—C57	1.476 (9)
C6—C25	1.492 (9)	C39—C40	1.426 (9)
C7—C8	1.417 (9)	C39—C60	1.487 (10)
C7—C28	1.516 (9)	C40—C41	1.438 (8)
C8—C9	1.374 (9)	C41—C42	1.426 (8)
C9—C10	1.389 (9)	C42—C43	1.362 (9)
C9—H9	0.9300	C42—C61	1.518 (9)
C10—C11	1.419 (8)	C43—C44	1.416 (8)
C11—C12	1.393 (9)	C43—C64	1.502 (9)
C11—C29	1.484 (8)	C44—C45	1.394 (9)
C12—C30	1.444 (9)	C45—C46	1.352 (8)
C12—C13	1.452 (8)	C45—H45	0.9300
C13—C14	1.407 (9)	C46—C47	1.436 (9)
C14—C15	1.375 (9)	C47—C48	1.353 (8)
C14—H14	0.9300	C47—C65	1.477 (8)
C15—C16	1.357 (8)	C48—C66	1.465 (10)
C15—H15	0.9300	C48—C49	1.470 (8)
C16—C17	1.462 (8)	C49—C50	1.393 (9)
C17—C18	1.374 (8)	C50—C51	1.368 (9)
C17—C33	1.460 (9)	C50—H50	0.9300
C18—C19	1.447 (9)	C51—C52	1.405 (10)
C18—C36	1.496 (8)	C51—H51	0.9300
C19—C20	1.370 (8)	C52—C53	1.447 (8)
C20—H20	0.9300	C53—C54	1.366 (10)
C21—C22B	1.42 (7)	C53—C69	1.472 (10)
C21—C22A	1.507 (19)	C54—C55	1.441 (9)
C21—H21A	0.9700	C54—C72	1.494 (9)
C21—H21B	0.9700	C55—C56	1.375 (9)
C22A—C23A	1.41 (2)	C56—H56	0.9300
C22A—H22A	0.9700	C57—C58	1.506 (12)
C22A—H22B	0.9700	C57—H57A	0.9700
C23A—C24	1.512 (15)	C57—H57B	0.9700
C23A—H23A	0.9700	C58—C59	1.455 (14)
C23A—H23B	0.9700	C58—H58A	0.9700
C22B—C23B	1.24 (6)	C58—H58B	0.9700
C22B—H22C	0.9700	C59—C60	1.501 (12)
C22B—H22D	0.9700	C59—H59A	0.9700
C23B—C24	1.62 (3)	C59—H59B	0.9700
C23B—H23C	0.9700	C60—H60A	0.9700
C23B—H23D	0.9700	C60—H60B	0.9700
C24—H24A	0.9700	C61—C62	1.513 (11)
C24—H24B	0.9700	C61—H61A	0.9700
C25—C26A	1.487 (19)	C61—H61B	0.9700
C25—C26B	1.54 (2)	C62—C63	1.376 (14)
C25—H25A	0.9700	C62—H62A	0.9700
C25—H25B	0.9700	C62—H62B	0.9700
C26A—C27	1.54 (3)	C63—C64	1.482 (12)
C26A—H26A	0.9700	C63—H63A	0.9700
C26A—H26B	0.9700	C63—H63B	0.9700
C26B—C27	1.36 (2)	C64—H64A	0.9700
C26B—H26C	0.9700	C64—H64B	0.9700
C26B—H26D	0.9700	C65—H65A	0.9600
C27—C28	1.468 (13)	C65—H65B	0.9600
C27—H27A	0.9700	C65—H65C	0.9600
C27—H27B	0.9700	C67—C68	1.488 (12)
C28—H28A	0.9700	C67—H67A	0.9700
C28—H28B	0.9700	C67—H67B	0.9700
C29—H29A	0.9600	C68—H68A	0.9600
C29—H29B	0.9600	C68—H68B	0.9600
C29—H29C	0.9600	C68—H68C	0.9600
C31—C32	1.451 (14)	C70—C71	1.444 (16)
C31—H31A	0.9700	C70—H70A	0.9700
C31—H31B	0.9700	C70—H70B	0.9700
C32—H32A	0.9600	C71—H71A	0.9600
C32—H32B	0.9600	C71—H71B	0.9600
C32—H32C	0.9600	C71—H71C	0.9600
C34—C35	1.479 (11)	C72—H72A	0.9600
C34—H34A	0.9700	C72—H72B	0.9600
C34—H34B	0.9700	C72—H72C	0.9600
C35—H35A	0.9600		
			
N2—Fe1—N1	74.2 (2)	O3—C34—H34B	110.5
N2—Fe1—N3	85.2 (2)	C35—C34—H34B	110.5
N1—Fe1—N3	151.4 (2)	H34A—C34—H34B	108.7
N2—Fe1—N4	151.50 (19)	C34—C35—H35A	109.5
N1—Fe1—N4	85.14 (19)	C34—C35—H35B	109.5
N3—Fe1—N4	105.96 (18)	H35A—C35—H35B	109.5
N2—Fe1—Cl1	103.37 (14)	C34—C35—H35C	109.5
N1—Fe1—Cl1	104.82 (14)	H35A—C35—H35C	109.5
N3—Fe1—Cl1	98.94 (13)	H35B—C35—H35C	109.5
N4—Fe1—Cl1	100.71 (14)	C18—C36—H36A	109.5
C4—N1—C1	107.7 (5)	C18—C36—H36B	109.5
C4—N1—Fe1	119.4 (4)	H36A—C36—H36B	109.5
C1—N1—Fe1	132.4 (5)	C18—C36—H36C	109.5
C5—N2—C8	105.6 (5)	H36A—C36—H36C	109.5
C5—N2—Fe1	120.0 (4)	H36B—C36—H36C	109.5
C8—N2—Fe1	133.6 (4)	N6—Fe2—N5	74.0 (2)
C13—N3—C10	106.5 (5)	N6—Fe2—N8	148.6 (2)
C13—N3—Fe1	126.9 (4)	N5—Fe2—N8	85.1 (2)
C10—N3—Fe1	125.4 (4)	N6—Fe2—N7	84.9 (2)
C16—N4—C19	107.6 (5)	N5—Fe2—N7	150.54 (19)
C16—N4—Fe1	125.8 (4)	N8—Fe2—N7	104.2 (2)
C19—N4—Fe1	124.9 (4)	N6—Fe2—Cl2	105.97 (14)
C30—O1—C31	120.2 (6)	N5—Fe2—Cl2	104.88 (14)
C33—O3—C34	117.3 (6)	N8—Fe2—Cl2	101.85 (14)
C20—C1—N1	121.0 (6)	N7—Fe2—Cl2	100.50 (14)
C20—C1—C2	130.1 (6)	C37—N5—C40	107.8 (5)
N1—C1—C2	108.9 (6)	C37—N5—Fe2	132.0 (4)
C3—C2—C1	106.9 (5)	C40—N5—Fe2	120.0 (4)
C3—C2—C21	124.2 (6)	C41—N6—C44	105.3 (5)
C1—C2—C21	128.8 (7)	C41—N6—Fe2	121.2 (4)
C2—C3—C4	106.4 (6)	C44—N6—Fe2	133.3 (4)
C2—C3—C24	122.0 (6)	C49—N7—C46	107.4 (5)
C4—C3—C24	131.7 (7)	C49—N7—Fe2	127.4 (4)
N1—C4—C3	110.2 (6)	C46—N7—Fe2	123.9 (4)
N1—C4—C5	112.5 (5)	C52—N8—C55	105.1 (5)
C3—C4—C5	137.3 (6)	C52—N8—Fe2	129.7 (4)
N2—C5—C4	111.8 (6)	C55—N8—Fe2	124.3 (4)
N2—C5—C6	111.3 (5)	C66—O5—C67	120.5 (7)
C4—C5—C6	136.9 (6)	C69—O7—C70	118.6 (6)
C7—C6—C5	104.7 (6)	N5—C37—C56	121.5 (6)
C7—C6—C25	124.2 (7)	N5—C37—C38	108.3 (5)
C5—C6—C25	130.9 (6)	C56—C37—C38	130.0 (6)
C6—C7—C8	109.2 (6)	C39—C38—C37	108.3 (6)
C6—C7—C28	122.9 (7)	C39—C38—C57	122.8 (7)
C8—C7—C28	128.0 (6)	C37—C38—C57	128.8 (6)
C9—C8—N2	119.2 (6)	C38—C39—C40	105.6 (6)
C9—C8—C7	131.5 (6)	C38—C39—C60	124.1 (7)
N2—C8—C7	109.2 (5)	C40—C39—C60	130.4 (6)
C8—C9—C10	127.4 (6)	N5—C40—C39	109.9 (5)
C8—C9—H9	116.3	N5—C40—C41	111.4 (5)
C10—C9—H9	116.3	C39—C40—C41	138.5 (6)
C9—C10—N3	126.0 (5)	N6—C41—C42	110.7 (5)
C9—C10—C11	123.7 (6)	N6—C41—C40	112.5 (5)
N3—C10—C11	110.3 (6)	C42—C41—C40	136.8 (6)
C12—C11—C10	107.1 (5)	C43—C42—C41	106.5 (6)
C12—C11—C29	126.5 (6)	C43—C42—C61	123.0 (6)
C10—C11—C29	126.4 (6)	C41—C42—C61	130.5 (6)
C11—C12—C30	123.6 (5)	C42—C43—C44	107.2 (5)
C11—C12—C13	106.4 (5)	C42—C43—C64	124.2 (6)
C30—C12—C13	130.0 (6)	C44—C43—C64	128.4 (6)
N3—C13—C14	129.1 (6)	N6—C44—C45	119.6 (6)
N3—C13—C12	109.7 (6)	N6—C44—C43	110.2 (5)
C14—C13—C12	121.3 (6)	C45—C44—C43	130.1 (5)
C15—C14—C13	137.0 (7)	C46—C45—C44	126.9 (6)
C15—C14—H14	111.5	C46—C45—H45	116.5
C13—C14—H14	111.5	C44—C45—H45	116.5
C16—C15—C14	139.3 (7)	C45—C46—N7	127.1 (6)
C16—C15—H15	110.3	C45—C46—C47	124.2 (6)
C14—C15—H15	110.3	N7—C46—C47	108.7 (5)
C15—C16—N4	129.7 (6)	C48—C47—C46	107.6 (5)
C15—C16—C17	121.7 (6)	C48—C47—C65	128.3 (6)
N4—C16—C17	108.5 (5)	C46—C47—C65	124.1 (6)
C18—C17—C33	127.5 (6)	C47—C48—C66	128.0 (6)
C18—C17—C16	107.7 (5)	C47—C48—C49	107.8 (6)
C33—C17—C16	124.8 (5)	C66—C48—C49	124.2 (6)
C17—C18—C19	106.6 (5)	N7—C49—C50	130.3 (6)
C17—C18—C36	130.5 (6)	N7—C49—C48	108.4 (5)
C19—C18—C36	122.9 (6)	C50—C49—C48	121.0 (6)
C20—C19—N4	127.4 (6)	C51—C50—C49	137.7 (7)
C20—C19—C18	122.9 (5)	C51—C50—H50	111.2
N4—C19—C18	109.7 (5)	C49—C50—H50	111.2
C1—C20—C19	126.4 (6)	C50—C51—C52	137.6 (7)
C1—C20—H20	116.8	C50—C51—H51	111.2
C19—C20—H20	116.8	C52—C51—H51	111.2
C22B—C21—C2	107 (2)	N8—C52—C51	128.6 (6)
C2—C21—C22A	113.2 (11)	N8—C52—C53	110.4 (6)
C22B—C21—H21A	126.2	C51—C52—C53	121.0 (6)
C2—C21—H21A	108.9	C54—C53—C52	107.5 (6)
C22A—C21—H21A	108.9	C54—C53—C69	123.5 (6)
C22B—C21—H21B	96.4	C52—C53—C69	129.0 (7)
C2—C21—H21B	108.9	C53—C54—C55	106.7 (6)
C22A—C21—H21B	108.9	C53—C54—C72	128.5 (6)
H21A—C21—H21B	107.8	C55—C54—C72	124.8 (7)
C23A—C22A—C21	120.3 (18)	C56—C55—N8	126.9 (5)
C23A—C22A—H22A	107.3	C56—C55—C54	122.8 (6)
C21—C22A—H22A	107.3	N8—C55—C54	110.2 (6)
C23A—C22A—H22B	107.3	C37—C56—C55	126.2 (6)
C21—C22A—H22B	107.3	C37—C56—H56	116.9
H22A—C22A—H22B	106.9	C55—C56—H56	116.9
C22A—C23A—C24	119.4 (12)	C38—C57—C58	112.9 (7)
C22A—C23A—H23A	107.5	C38—C57—H57A	109.0
C24—C23A—H23A	107.5	C58—C57—H57A	109.0
C22A—C23A—H23B	107.5	C38—C57—H57B	109.0
C24—C23A—H23B	107.5	C58—C57—H57B	109.0
H23A—C23A—H23B	107.0	H57A—C57—H57B	107.8
C23B—C22B—C21	133 (4)	C59—C58—C57	116.1 (9)
C23B—C22B—H22C	104.0	C59—C58—H58A	108.3
C21—C22B—H22C	104.0	C57—C58—H58A	108.3
C23B—C22B—H22D	104.0	C59—C58—H58B	108.3
C21—C22B—H22D	104.0	C57—C58—H58B	108.3
H22C—C22B—H22D	105.5	H58A—C58—H58B	107.4
C22B—C23B—C24	119 (3)	C58—C59—C60	117.2 (9)
C22B—C23B—H23C	107.5	C58—C59—H59A	108.0
C24—C23B—H23C	107.5	C60—C59—H59A	108.0
C22B—C23B—H23D	107.5	C58—C59—H59B	108.0
C24—C23B—H23D	107.5	C60—C59—H59B	108.0
H23C—C23B—H23D	107.0	H59A—C59—H59B	107.3
C3—C24—C23A	112.8 (8)	C39—C60—C59	111.8 (7)
C3—C24—C23B	105.9 (10)	C39—C60—H60A	109.3
C3—C24—H24A	109.0	C59—C60—H60A	109.3
C23A—C24—H24A	109.0	C39—C60—H60B	109.3
C23B—C24—H24A	82.2	C59—C60—H60B	109.3
C3—C24—H24B	109.0	H60A—C60—H60B	107.9
C23A—C24—H24B	109.0	C62—C61—C42	109.6 (7)
C23B—C24—H24B	137.5	C62—C61—H61A	109.8
H24A—C24—H24B	107.8	C42—C61—H61A	109.8
C26A—C25—C6	111.7 (9)	C62—C61—H61B	109.8
C6—C25—C26B	110.8 (10)	C42—C61—H61B	109.8
C26A—C25—H25A	109.3	H61A—C61—H61B	108.2
C6—C25—H25A	109.3	C63—C62—C61	121.7 (9)
C26B—C25—H25A	80.4	C63—C62—H62A	106.9
C26A—C25—H25B	109.3	C61—C62—H62A	106.9
C6—C25—H25B	109.3	C63—C62—H62B	106.9
C26B—C25—H25B	133.1	C61—C62—H62B	106.9
H25A—C25—H25B	108.0	H62A—C62—H62B	106.7
C25—C26A—C27	112.3 (15)	C62—C63—C64	119.7 (8)
C25—C26A—H26A	109.1	C62—C63—H63A	107.4
C27—C26A—H26A	109.1	C64—C63—H63A	107.4
C25—C26A—H26B	109.1	C62—C63—H63B	107.4
C27—C26A—H26B	109.1	C64—C63—H63B	107.4
H26A—C26A—H26B	107.9	H63A—C63—H63B	106.9
C27—C26B—C25	120.3 (18)	C63—C64—C43	112.2 (7)
C27—C26B—H26C	107.2	C63—C64—H64A	109.2
C25—C26B—H26C	107.2	C43—C64—H64A	109.2
C27—C26B—H26D	107.2	C63—C64—H64B	109.2
C25—C26B—H26D	107.2	C43—C64—H64B	109.2
H26C—C26B—H26D	106.9	H64A—C64—H64B	107.9
C26B—C27—C28	125.1 (12)	C47—C65—H65A	109.5
C28—C27—C26A	117.6 (13)	C47—C65—H65B	109.5
C26B—C27—H27A	75.9	H65A—C65—H65B	109.5
C28—C27—H27A	107.9	C47—C65—H65C	109.5
C26A—C27—H27A	107.9	H65A—C65—H65C	109.5
C26B—C27—H27B	123.6	H65B—C65—H65C	109.5
C28—C27—H27B	107.9	O6—C66—O5	119.5 (8)
C26A—C27—H27B	107.9	O6—C66—C48	128.1 (7)
H27A—C27—H27B	107.2	O5—C66—C48	112.4 (6)
C27—C28—C7	112.2 (7)	O5—C67—C68	105.9 (8)
C27—C28—H28A	109.2	O5—C67—H67A	110.6
C7—C28—H28A	109.2	C68—C67—H67A	110.6
C27—C28—H28B	109.2	O5—C67—H67B	110.6
C7—C28—H28B	109.2	C68—C67—H67B	110.6
H28A—C28—H28B	107.9	H67A—C67—H67B	108.7
C11—C29—H29A	109.5	C67—C68—H68A	109.5
C11—C29—H29B	109.5	C67—C68—H68B	109.5
H29A—C29—H29B	109.5	H68A—C68—H68B	109.5
C11—C29—H29C	109.5	C67—C68—H68C	109.5
H29A—C29—H29C	109.5	H68A—C68—H68C	109.5
H29B—C29—H29C	109.5	H68B—C68—H68C	109.5
O2—C30—O1	117.3 (8)	O8—C69—O7	122.9 (7)
O2—C30—C12	124.8 (8)	O8—C69—C53	123.0 (8)
O1—C30—C12	117.9 (6)	O7—C69—C53	114.1 (6)
O1—C31—C32	108.6 (7)	C71—C70—O7	110.1 (8)
O1—C31—H31A	110.0	C71—C70—H70A	109.6
C32—C31—H31A	110.0	O7—C70—H70A	109.6
O1—C31—H31B	110.0	C71—C70—H70B	109.6
C32—C31—H31B	110.0	O7—C70—H70B	109.6
H31A—C31—H31B	108.3	H70A—C70—H70B	108.2
C31—C32—H32A	109.5	C70—C71—H71A	109.5
C31—C32—H32B	109.5	C70—C71—H71B	109.5
H32A—C32—H32B	109.5	H71A—C71—H71B	109.5
C31—C32—H32C	109.5	C70—C71—H71C	109.5
H32A—C32—H32C	109.5	H71A—C71—H71C	109.5
H32B—C32—H32C	109.5	H71B—C71—H71C	109.5
O4—C33—O3	120.1 (6)	C54—C72—H72A	109.5
O4—C33—C17	128.8 (6)	C54—C72—H72B	109.5
O3—C33—C17	111.1 (5)	H72A—C72—H72B	109.5
O3—C34—C35	106.0 (7)	C54—C72—H72C	109.5
O3—C34—H34A	110.5	H72A—C72—H72C	109.5
C35—C34—H34A	110.5	H72B—C72—H72C	109.5
			
N2—Fe1—N1—C4	−12.8 (4)	C11—C12—C30—O1	159.8 (6)
N3—Fe1—N1—C4	−58.1 (6)	C13—C12—C30—O1	−21.4 (10)
N4—Fe1—N1—C4	−173.0 (4)	C30—O1—C31—C32	−174.8 (9)
Cl1—Fe1—N1—C4	87.2 (4)	C34—O3—C33—O4	−0.3 (9)
N2—Fe1—N1—C1	177.5 (5)	C34—O3—C33—C17	−178.5 (5)
N3—Fe1—N1—C1	132.1 (5)	C18—C17—C33—O4	166.4 (7)
N4—Fe1—N1—C1	17.3 (5)	C16—C17—C33—O4	−13.0 (11)
Cl1—Fe1—N1—C1	−82.5 (5)	C18—C17—C33—O3	−15.6 (8)
N1—Fe1—N2—C5	13.1 (4)	C16—C17—C33—O3	165.0 (5)
N3—Fe1—N2—C5	173.1 (4)	C33—O3—C34—C35	−172.6 (6)
N4—Fe1—N2—C5	58.1 (6)	N6—Fe2—N5—C37	176.2 (5)
Cl1—Fe1—N2—C5	−88.8 (4)	N8—Fe2—N5—C37	19.9 (5)
N1—Fe1—N2—C8	−179.5 (5)	N7—Fe2—N5—C37	130.3 (5)
N3—Fe1—N2—C8	−19.4 (5)	Cl2—Fe2—N5—C37	−81.1 (5)
N4—Fe1—N2—C8	−134.4 (5)	N6—Fe2—N5—C40	−8.5 (4)
Cl1—Fe1—N2—C8	78.7 (5)	N8—Fe2—N5—C40	−164.8 (4)
N2—Fe1—N3—C13	−178.5 (5)	N7—Fe2—N5—C40	−54.4 (6)
N1—Fe1—N3—C13	−135.2 (5)	Cl2—Fe2—N5—C40	94.2 (4)
N4—Fe1—N3—C13	−25.2 (5)	N5—Fe2—N6—C41	7.5 (4)
Cl1—Fe1—N3—C13	78.7 (4)	N8—Fe2—N6—C41	57.7 (6)
N2—Fe1—N3—C10	15.5 (4)	N7—Fe2—N6—C41	166.7 (4)
N1—Fe1—N3—C10	58.9 (6)	Cl2—Fe2—N6—C41	−93.8 (4)
N4—Fe1—N3—C10	168.8 (4)	N5—Fe2—N6—C44	−179.6 (5)
Cl1—Fe1—N3—C10	−87.3 (4)	N8—Fe2—N6—C44	−129.4 (5)
N2—Fe1—N4—C16	137.2 (4)	N7—Fe2—N6—C44	−20.3 (5)
N1—Fe1—N4—C16	−179.7 (4)	Cl2—Fe2—N6—C44	79.1 (5)
N3—Fe1—N4—C16	27.1 (5)	N6—Fe2—N7—C49	−174.1 (5)
Cl1—Fe1—N4—C16	−75.5 (4)	N5—Fe2—N7—C49	−130.2 (5)
N2—Fe1—N4—C19	−59.8 (6)	N8—Fe2—N7—C49	−24.6 (5)
N1—Fe1—N4—C19	−16.8 (4)	Cl2—Fe2—N7—C49	80.6 (5)
N3—Fe1—N4—C19	−169.9 (4)	N6—Fe2—N7—C46	20.4 (4)
Cl1—Fe1—N4—C19	87.4 (4)	N5—Fe2—N7—C46	64.3 (6)
C4—N1—C1—C20	178.2 (5)	N8—Fe2—N7—C46	169.8 (4)
Fe1—N1—C1—C20	−11.2 (8)	Cl2—Fe2—N7—C46	−85.0 (4)
C4—N1—C1—C2	−0.7 (6)	N6—Fe2—N8—C52	125.0 (5)
Fe1—N1—C1—C2	169.9 (4)	N5—Fe2—N8—C52	172.9 (5)
C20—C1—C2—C3	−177.4 (6)	N7—Fe2—N8—C52	21.2 (5)
N1—C1—C2—C3	1.4 (6)	Cl2—Fe2—N8—C52	−82.9 (5)
C20—C1—C2—C21	−1.4 (10)	N6—Fe2—N8—C55	−67.3 (6)
N1—C1—C2—C21	177.4 (6)	N5—Fe2—N8—C55	−19.5 (4)
C1—C2—C3—C4	−1.5 (6)	N7—Fe2—N8—C55	−171.1 (4)
C21—C2—C3—C4	−177.8 (5)	Cl2—Fe2—N8—C55	84.7 (4)
C1—C2—C3—C24	178.0 (5)	C40—N5—C37—C56	173.3 (5)
C21—C2—C3—C24	1.7 (9)	Fe2—N5—C37—C56	−11.0 (8)
C1—N1—C4—C3	−0.3 (6)	C40—N5—C37—C38	−1.8 (6)
Fe1—N1—C4—C3	−172.3 (4)	Fe2—N5—C37—C38	173.9 (4)
C1—N1—C4—C5	−177.1 (4)	N5—C37—C38—C39	2.0 (6)
Fe1—N1—C4—C5	10.8 (6)	C56—C37—C38—C39	−172.5 (6)
C2—C3—C4—N1	1.1 (6)	N5—C37—C38—C57	178.8 (6)
C24—C3—C4—N1	−178.3 (6)	C56—C37—C38—C57	4.3 (10)
C2—C3—C4—C5	176.9 (6)	C37—C38—C39—C40	−1.4 (6)
C24—C3—C4—C5	−2.5 (11)	C57—C38—C39—C40	−178.4 (6)
C8—N2—C5—C4	178.0 (4)	C37—C38—C39—C60	178.8 (5)
Fe1—N2—C5—C4	−11.4 (6)	C57—C38—C39—C60	1.8 (9)
C8—N2—C5—C6	−1.5 (6)	C37—N5—C40—C39	1.0 (6)
Fe1—N2—C5—C6	169.1 (4)	Fe2—N5—C40—C39	−175.3 (3)
N1—C4—C5—N2	0.3 (7)	C37—N5—C40—C41	−175.5 (4)
C3—C4—C5—N2	−175.4 (6)	Fe2—N5—C40—C41	8.2 (6)
N1—C4—C5—C6	179.6 (6)	C38—C39—C40—N5	0.3 (6)
C3—C4—C5—C6	3.9 (12)	C60—C39—C40—N5	−179.9 (6)
N2—C5—C6—C7	0.6 (6)	C38—C39—C40—C41	175.3 (6)
C4—C5—C6—C7	−178.7 (6)	C60—C39—C40—C41	−4.9 (11)
N2—C5—C6—C25	−175.5 (6)	C44—N6—C41—C42	0.8 (6)
C4—C5—C6—C25	5.1 (11)	Fe2—N6—C41—C42	175.5 (3)
C5—C6—C7—C8	0.5 (6)	C44—N6—C41—C40	179.8 (4)
C25—C6—C7—C8	177.0 (5)	Fe2—N6—C41—C40	−5.5 (6)
C5—C6—C7—C28	−179.6 (5)	N5—C40—C41—N6	−1.7 (6)
C25—C6—C7—C28	−3.1 (9)	C39—C40—C41—N6	−176.7 (6)
C5—N2—C8—C9	−177.5 (5)	N5—C40—C41—C42	176.9 (6)
Fe1—N2—C8—C9	13.7 (8)	C39—C40—C41—C42	1.9 (11)
C5—N2—C8—C7	1.8 (6)	N6—C41—C42—C43	−1.9 (6)
Fe1—N2—C8—C7	−166.9 (4)	C40—C41—C42—C43	179.4 (6)
C6—C7—C8—C9	177.7 (6)	N6—C41—C42—C61	177.3 (5)
C28—C7—C8—C9	−2.1 (10)	C40—C41—C42—C61	−1.3 (10)
C6—C7—C8—N2	−1.5 (6)	C41—C42—C43—C44	2.2 (6)
C28—C7—C8—N2	178.6 (5)	C61—C42—C43—C44	−177.2 (5)
N2—C8—C9—C10	2.9 (9)	C41—C42—C43—C64	178.7 (5)
C7—C8—C9—C10	−176.3 (6)	C61—C42—C43—C64	−0.6 (9)
C8—C9—C10—N3	−4.7 (9)	C41—N6—C44—C45	−174.9 (5)
C8—C9—C10—C11	176.3 (5)	Fe2—N6—C44—C45	11.3 (8)
C13—N3—C10—C9	−176.9 (5)	C41—N6—C44—C43	0.6 (6)
Fe1—N3—C10—C9	−8.6 (8)	Fe2—N6—C44—C43	−173.2 (4)
C13—N3—C10—C11	2.2 (6)	C42—C43—C44—N6	−1.8 (6)
Fe1—N3—C10—C11	170.5 (4)	C64—C43—C44—N6	−178.1 (5)
C9—C10—C11—C12	178.2 (5)	C42—C43—C44—C45	173.1 (6)
N3—C10—C11—C12	−0.9 (6)	C64—C43—C44—C45	−3.3 (10)
C9—C10—C11—C29	−0.3 (9)	N6—C44—C45—C46	5.6 (9)
N3—C10—C11—C29	−179.4 (5)	C43—C44—C45—C46	−168.9 (6)
C10—C11—C12—C30	178.4 (5)	C44—C45—C46—N7	−2.7 (9)
C29—C11—C12—C30	−3.0 (9)	C44—C45—C46—C47	174.5 (5)
C10—C11—C12—C13	−0.7 (6)	C49—N7—C46—C45	177.0 (5)
C29—C11—C12—C13	177.9 (5)	Fe2—N7—C46—C45	−15.0 (7)
C10—N3—C13—C14	177.3 (6)	C49—N7—C46—C47	−0.5 (6)
Fe1—N3—C13—C14	9.2 (9)	Fe2—N7—C46—C47	167.5 (3)
C10—N3—C13—C12	−2.6 (6)	C45—C46—C47—C48	−176.4 (5)
Fe1—N3—C13—C12	−170.7 (3)	N7—C46—C47—C48	1.2 (6)
C11—C12—C13—N3	2.0 (6)	C45—C46—C47—C65	3.2 (9)
C30—C12—C13—N3	−177.0 (6)	N7—C46—C47—C65	−179.2 (5)
C11—C12—C13—C14	−177.8 (5)	C46—C47—C48—C66	179.0 (6)
C30—C12—C13—C14	3.2 (9)	C65—C47—C48—C66	−0.6 (11)
N3—C13—C14—C15	6.2 (12)	C46—C47—C48—C49	−1.4 (6)
C12—C13—C14—C15	−174.0 (7)	C65—C47—C48—C49	179.0 (6)
C13—C14—C15—C16	3.4 (15)	C46—N7—C49—C50	−173.9 (6)
C14—C15—C16—N4	−8.5 (12)	Fe2—N7—C49—C50	18.6 (9)
C14—C15—C16—C17	175.2 (7)	C46—N7—C49—C48	−0.4 (6)
C19—N4—C16—C15	−177.3 (6)	Fe2—N7—C49—C48	−167.8 (4)
Fe1—N4—C16—C15	−11.9 (8)	C47—C48—C49—N7	1.1 (7)
C19—N4—C16—C17	−0.6 (6)	C66—C48—C49—N7	−179.2 (6)
Fe1—N4—C16—C17	164.7 (3)	C47—C48—C49—C50	175.4 (6)
C15—C16—C17—C18	177.3 (5)	C66—C48—C49—C50	−5.0 (10)
N4—C16—C17—C18	0.4 (6)	N7—C49—C50—C51	−4.0 (14)
C15—C16—C17—C33	−3.2 (8)	C48—C49—C50—C51	−176.8 (8)
N4—C16—C17—C33	179.9 (5)	C49—C50—C51—C52	0.1 (16)
C33—C17—C18—C19	−179.4 (5)	C55—N8—C52—C51	178.9 (6)
C16—C17—C18—C19	0.1 (6)	Fe2—N8—C52—C51	−11.6 (9)
C33—C17—C18—C36	0.2 (10)	C55—N8—C52—C53	1.7 (6)
C16—C17—C18—C36	179.8 (5)	Fe2—N8—C52—C53	171.2 (4)
C16—N4—C19—C20	178.3 (5)	C50—C51—C52—N8	−0.2 (14)
Fe1—N4—C19—C20	12.8 (8)	C50—C51—C52—C53	176.8 (8)
C16—N4—C19—C18	0.7 (6)	N8—C52—C53—C54	−2.4 (7)
Fe1—N4—C19—C18	−164.8 (3)	C51—C52—C53—C54	−179.9 (6)
C17—C18—C19—C20	−178.2 (5)	N8—C52—C53—C69	176.9 (6)
C36—C18—C19—C20	2.1 (8)	C51—C52—C53—C69	−0.5 (10)
C17—C18—C19—N4	−0.5 (6)	C52—C53—C54—C55	2.1 (7)
C36—C18—C19—N4	179.8 (5)	C69—C53—C54—C55	−177.3 (6)
N1—C1—C20—C19	−2.1 (9)	C52—C53—C54—C72	−177.0 (6)
C2—C1—C20—C19	176.6 (5)	C69—C53—C54—C72	3.6 (11)
N4—C19—C20—C1	0.5 (9)	C52—N8—C55—C56	−176.4 (5)
C18—C19—C20—C1	177.9 (5)	Fe2—N8—C55—C56	13.4 (8)
C3—C2—C21—C22B	21 (2)	C52—N8—C55—C54	−0.3 (6)
C1—C2—C21—C22B	−155 (2)	Fe2—N8—C55—C54	−170.6 (4)
C3—C2—C21—C22A	2.5 (15)	C53—C54—C55—C56	175.1 (5)
C1—C2—C21—C22A	−172.9 (14)	C72—C54—C55—C56	−5.8 (9)
C22B—C21—C22A—C23A	−95 (8)	C53—C54—C55—N8	−1.1 (7)
C2—C21—C22A—C23A	−21 (3)	C72—C54—C55—N8	178.0 (5)
C21—C22A—C23A—C24	35 (3)	N5—C37—C56—C55	−6.0 (9)
C2—C21—C22B—C23B	−20 (6)	C38—C37—C56—C55	167.9 (6)
C22A—C21—C22B—C23B	93 (10)	N8—C55—C56—C37	4.1 (9)
C21—C22B—C23B—C24	−4(8)	C54—C55—C56—C37	−171.4 (5)
C2—C3—C24—C23A	10.5 (11)	C39—C38—C57—C58	10.1 (10)
C4—C3—C24—C23A	−170.2 (9)	C37—C38—C57—C58	−166.2 (8)
C2—C3—C24—C23B	−23.7 (14)	C38—C57—C58—C59	−34.2 (13)
C4—C3—C24—C23B	155.6 (13)	C57—C58—C59—C60	47.8 (15)
C22A—C23A—C24—C3	−29 (2)	C38—C39—C60—C59	9.2 (10)
C22A—C23A—C24—C23B	55 (2)	C40—C39—C60—C59	−170.6 (7)
C22B—C23B—C24—C3	25 (4)	C58—C59—C60—C39	−33.6 (13)
C22B—C23B—C24—C23A	−82 (4)	C43—C42—C61—C62	11.2 (9)
C7—C6—C25—C26A	−18.4 (18)	C41—C42—C61—C62	−167.9 (7)
C5—C6—C25—C26A	157.1 (17)	C42—C61—C62—C63	−30.7 (13)
C7—C6—C25—C26B	16 (2)	C61—C62—C63—C64	39.5 (16)
C5—C6—C25—C26B	−169 (2)	C62—C63—C64—C43	−24.4 (13)
C6—C25—C26A—C27	43 (3)	C42—C43—C64—C63	5.7 (9)
C26B—C25—C26A—C27	−52 (2)	C44—C43—C64—C63	−178.5 (7)
C26A—C25—C26B—C27	73 (4)	C67—O5—C66—O6	2.8 (14)
C6—C25—C26B—C27	−25 (4)	C67—O5—C66—C48	−179.8 (7)
C25—C26B—C27—C28	22 (5)	C47—C48—C66—O6	164.4 (10)
C25—C26B—C27—C26A	−65 (3)	C49—C48—C66—O6	−15.2 (14)
C25—C26A—C27—C26B	61 (2)	C47—C48—C66—O5	−12.8 (11)
C25—C26A—C27—C28	−52 (3)	C49—C48—C66—O5	167.7 (6)
C26B—C27—C28—C7	−8(3)	C66—O5—C67—C68	−165.2 (8)
C26A—C27—C28—C7	29.7 (16)	C70—O7—C69—O8	2.8 (11)
C6—C7—C28—C27	−2.6 (10)	C70—O7—C69—C53	−176.5 (6)
C8—C7—C28—C27	177.3 (7)	C54—C53—C69—O8	35.6 (11)
C31—O1—C30—O2	0.2 (11)	C52—C53—C69—O8	−143.6 (8)
C31—O1—C30—C12	−179.6 (7)	C54—C53—C69—O7	−145.0 (6)
C11—C12—C30—O2	−20.0 (11)	C52—C53—C69—O7	35.7 (10)
C13—C12—C30—O2	158.9 (8)	C69—O7—C70—C71	159.7 (10)
